# PP2Ac upregulates PI3K-Akt signaling and induces hepatocyte apoptosis in liver donor after brain death

**DOI:** 10.1007/s10495-019-01570-8

**Published:** 2019-10-11

**Authors:** Yan Xiong, Jianan Lan, Kaixin Huang, Yaruo Zhang, Lewei Zheng, Yanfeng Wang, Qifa Ye

**Affiliations:** 1 Zhongnan Hospital of Wuhan University, Institute of Hepatobiliary Diseases of Wuhan University, Transplant Center of Wuhan University, Hubei Key Laboratory of Medical Technology on Transplantation, Wuhan, 430071 China; 2 The 3rd Xiangya Hospital of Central South University, Research Center of National Health Ministry on Transplantation Medicine Engineering and Technology, Changsha, 410013 China; 3 Division of Transplant Immunology, Department of Pathology and Laboratory Medicine, Children’s Hospital of Philadelphia and Perelman School of Medicine at the University of Pennsylvania, Philadelphia, PA 19104 USA

**Keywords:** Donation after brain death, Hepatocyte damage, Apoptosis, PP2A

## Abstract

Multiple research groups have demonstrated that the outcome of patients receiving liver grafts from brain death donors (DBD) is poorer when compared with patients receiving grafts from living donors. This might be due to an increased hepatocyte apoptosis induced after brain death (BD). In this work, we found that the activity of PP2A-Akt pathway is significantly increased in clinical donor ex vivo hepatocytes after BD by iTRAQ protein quantification analysis. The same results were confirmed in animal models. A time-dependent promotion of apoptosis was also found in DBD rabbit liver, as demonstrated by the increased levels of cleaved Caspase 3 and the decreased of Bcl-2. To further investigate the roles of PP2A and Akt in regulating apoptosis of hepatocytes after BD, we cultivated human liver cell line L02 with serum deprivation and hypoxia, to simulate the ischemic and hypoxic conditions of hepatocytes in DBD. Increased apoptosis and decreased viability were observed during the time in this model. Meanwhile PP2A activity and Akt activity were respectively increased and decreased. Notably, the proportion of Akt phosphorylation at Ser473 decreased, while other known targets of PP2A (p38, JNK and ERK) were not affected in terms of protein levels or phosphorylation. These results suggested that PP2A is involved in apoptotic induction of hepatocytes after brain death by specific suppression of Akt. This discovery was further confirmed with pharmaceutical and genetic methods. Our work implied potential targets for reducing liver cell apoptosis and improving organ donor quality after BD.

**Electronic supplementary material:**

The online version of this article (10.1007/s10495-019-01570-8) contains supplementary material, which is available to authorized users.

## Introduction

There exists a vast disparity between the number of patients on transplant waiting list and that of available organs [1–4] and the main source of donor organs are from brain death (DBD) and cardiac death (DCD). Several kidney-transplant studies have revealed a consistent and significant difference in the graft survival between grafts derived from brain-dead (BD) donors and the grafts from living donors. There are more incidents of primary graft non function and poor graft survival when grafts were from BD patients than that from living donors. Animal studies showed the same tendency: BD causes deteriorated organ quality and viability for both kidney and liver [5–7]. It was hypothesized that BD has a detrimental effect on donor organ viability [8, 9].

Even though a number of studies have assessed the effects of BD on kidney and liver grafts viability, the mechanism by which BD induces kidney and liver injuries has not been fully clarified. It has been suggested that apoptosis after BD contributes strongly to organ injury [10–13]. But it is not clear yet how BD causes apoptosis.

PP2A, a serine/threonine protein phosphatases made up of three subunits A, B and C, is demonstrated to be involved in apoptosis. The subunit A is required for the formation of the trimeric complex, subunit C (PP2Ac) has catalytic domain [14] and B is the regulatory that binds with the AC core complex to form the heterotrimeric holoenzyme [15] and is responsible for the substrate specificity of PP2A [16]. PP2A can act on a wide range of substrates through the holoenzyme structure that contains a distinct B subunit from the families of the proteins: B (PR55), Bʹ (B56 or PR56), B″ (PR72) and B‴ (PR93/PR110) [17]. Some of the most common PP2A substrates include apoptotic protein Bax, DNA repair protein ATM, tumor suppressor protein pRb, receptor EGFR, MAPK cell signaling protein JNK and cell cycle-associated proteins CDK4, CDK9, CD16, CDC6 and CDC25 [16]. PP2A can not only indirectly regulate the induction of apoptosis via the dephosphorylation of cell signaling and tumor suppressor proteins such as p38, JNK, Akt and Rb respectively [16, 18], but can also act directly on apoptotic and anti-apoptotic proteins [19, 20]. For example, it has been shown that protein PKR promotes mitochondrial localization of the PP2A–PR61α complex leading to Bcl-2 dephosphorylation [19]. In addition, PP2A has been reported to dephosphorylate Bax and enhance its pro-apoptotic function via direct interaction with catalytic subunit PP2A/C [20].

Protein kinase B (PKB), also known as Akt, a serine/threonine-specific protein kinase that plays a key role in glucose metabolism, apoptosis, cell proliferation, transcription and cell migration, is mainly regulated by protein kinases and protein phosphatases [21–23]; both are highly expressed in the kidney, liver, and spleen. Akt activity has been shown to be regulated by PP2A. Overexpressed-eIF3I interacts with the activated form of oncogenic Akt1 via inhibition of PP2A phosphorylation in human hepatocellular carcinoma [24], whereas the protein REDD1 enhances PP2A-mediated dephosphorylation of Akt resulting in repression of mTORC1 signaling in 293T cells [25]. In addition, it has been shown that PP2A regulates p53 and Akt cooperatively leading to apoptosis evasion by neuron cells [26]. Lastly ceramide mediated vascular dysfunction in diet-induced obesity has been shown to be through dephosphorylation of the eNOS-Akt complex by PP2A [27]. Although the actions of PP2A and Akt have been well defined in the aforementioned studies, the regulation of Akt signaling in the cellular apoptosis in the donor liver from BD patients has not yet been reported.

Based on these previous findings, we suspected that BD may induce Akt inactivation and hepatocyte apoptosis through PP2A activation in donor liver from BD individuals. In the present study, we explored the effects of BD on hepatocyte apoptosis and possible link of Akt and PP2A with the apoptosis.

## Results

### Brain death (BD) inactivates Akt, activates PP2A and causes liver apoptosis both in animal model and donor liver

**Fig. 1:**
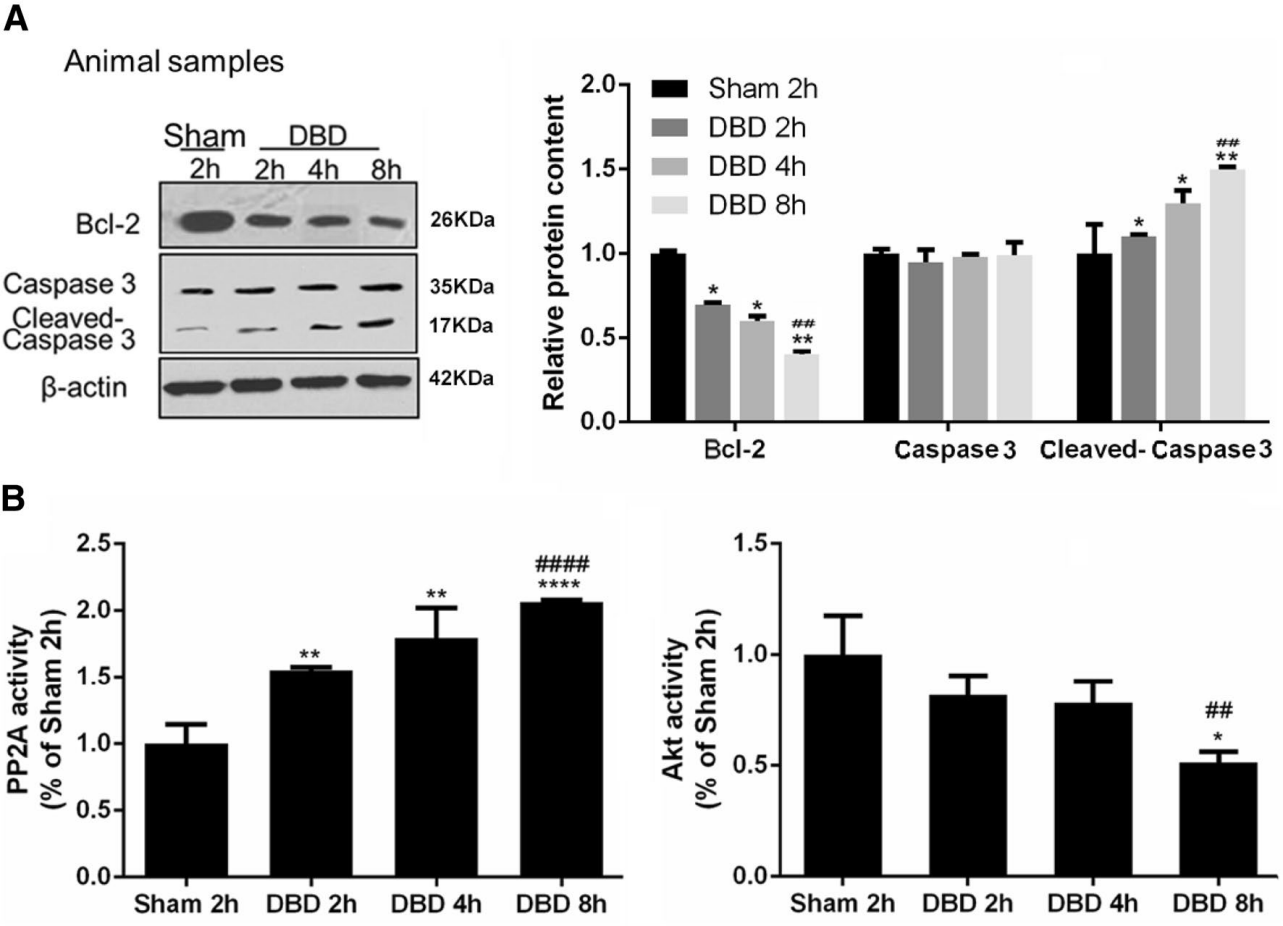
Increased apoptosis as well as PP2A activation and Akt inactivation were observed in liver tissues from donation after brain death (DBD) rabbit model. The samples were harvested at 2 h, 4 h, and 8 h post brain death or 2 h post sham operation. **a** The amount of apoptotic markers Bcl-2, Caspase-3 and cleaved Caspase 3 were measured by western blots. Left: representative images of western blots. Right: semi-quantitative analysis with β-actin as an internal control. The results were normalized to the Sham 2 h group. **b** The activities of PP2A and Akt were measured, as described in the “Materials and methods”. The results were normalized to the Sham 2 h group. All data were expressed as the mean ± SEM. n = 10 for each group. *p < 0.05, **p < 0.01, and ****p < 0.0001 versus the Sham 2 h group; ^##^*p* < 0.01, and ^####^*p* < 0.0001 versus the DBD 2 h group

**Fig. 2:**
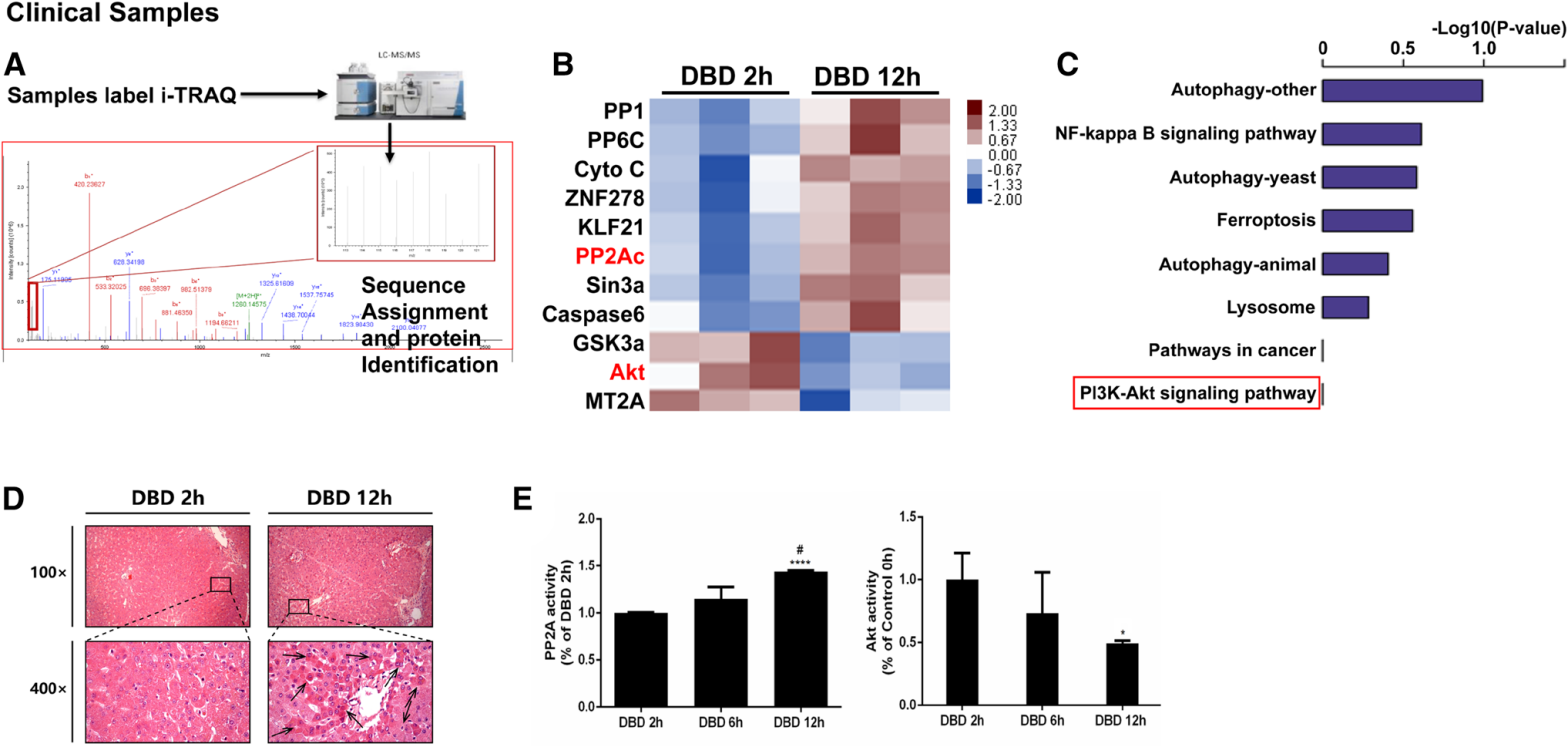
PP2A activation and Akt inactivation in clinical DBD samples were observed with different time span of brain death. **a** Quantitative mass spectrometry was used to analyze pathway (**b**) and differential proteins (**c**) between DBD 2 h and DBD 12 h. n = 3 for the DBD 2 h group and n = 3 for the DBD 12 h group, p value < 0.05. **d** Representative micrographs of the liver with apoptotic bodies (black arrows) are shown by H&E staining at a magnification of × 100 (uper) and × 400 (bottom). **e** The results were normalized to the DBD 2 h group. All data were expressed as the mean ± SEM. n = 9 for the DBD 2 h group, n = 5 for the DBD 6 h group and n = 6 for the DBD 12 h group. *p < 0.05 and ****p < 0.0001 versus the DBD 2 h group; #*p* < 0.05 versus the DBD 6 h group

We first observed an induction of apoptosis in liver tissues from the DBD animal model (Fig. 1a). Compared to the sham group, Bcl-2 levels were progressively downregulated in the DBD group from 2 to 8 h post brain death, while cleaved-Caspase 3 was significantly elevated with total caspase-3 remained stable. In the meantime, the activity of PP2A was significantly increased during the time in liver tissues in the DBD group while the activity of Akt showed an opposite trend (Fig. 1b). To investigate how the PP2A affect the liver apoptosis, quantitative mass spectrometry was used to analyze differentially expressed proteins in human donor liver cells collected 2 h or 12 h after brain death (Fig. 2a). Interestingly, Akt, PP2A and proteins belonging to PI3K-Akt pathway are enriched in these samples (Fig. 2b, c), suggesting that both PP2A and PI3K-Akt pathways are involved in the regulation of apoptosis in the liver tissues after brain death. Moreover, H&E staining of 4 donor livers evidenced more apoptotic bodies (Fig. 2d black arrow) in samples harvested 12 h after BD in respect to the ones collected after 2 h (Fig. 2d). Elisa assays performed on the same samples as in Fig. 2d confirmed the increased activity of PP2A and the decreased activity of Akt in samples collected 12 h after BD in respect to 2 h (Fig. 2e). These observations were in accordance with the results obtained from animal samples (Fig. 1).

### Hypoxia and ischemia (mimic BD) induced apoptosis is dependent on Akt inactivation and PP2A activation in vitro

Then we set up an in vitro model for further investigating the roles of PP2A and Akt in regulating apoptosis of hepatocytes after brain death. We cultivated human liver cell line L02 in starvation (with serum deprivation) and under hypoxia (low oxygen concentration), simulating the ischemic and hypoxic conditions of hepatocytes in BD patients [30].

**Fig. 3:**
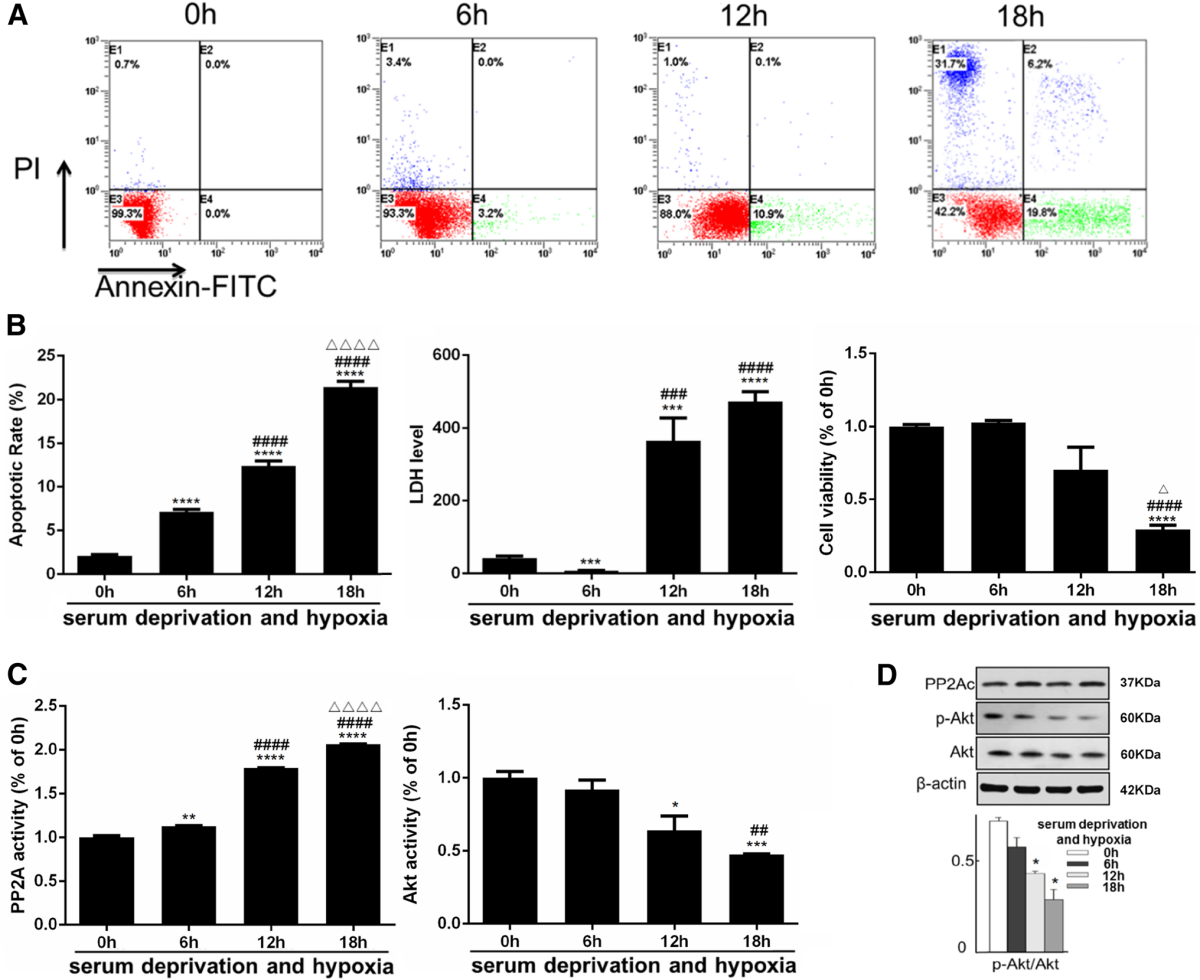
Increased apoptosis and impaired viability were observed together with PP2A activation and Akt inactivation in L02 cells cultivated with serum deprivation and hypoxia (simulating the ischemic and hypoxic conditions of hepatocytes in BD individuals). The measurements below were performed at 0, 6, 12 and 18 h exposure of serum deprivation and hypoxia. **a** Fluorescence-activated cell sorting (FACS) after Annexin V and PI double staining was used to detect the apoptosis rate. Representative FACS plots were shown with the right lower quadrant indicating apoptosis and the upper left quadrant indicating necrosis. **b** Left: quantitative analysis of the apoptotic rate. Middle: hepatocellular function reflected by lactate dehydrogenase (LDH) level; the unit: U/L. Right: cell viability measured with CCK8 assay. **c** The activities of PP2A and Akt were measured with the results normalized to the 2 h group. **d** The total amount and phosphorylation level at Ser473 of Akt together with total amount of PP2Ac were measured by western blots. Top: representative images of western blots. Bottom: Semi-quantitative analysis with β-actin as internal control. The results were normalized to the 2 h group. All data were expressed as the mean ± SEM. n ≥ 3 for each group in each measurement. *p < 0.05, **p < 0.01, ***p < 0.001 and ****p < 0.0001 versus the 0 h group; ^##^p < 0.01, ^###^p < 0.001, and ^####^p < 0.0001 versus the 6 h group; ^∆^p < 0.05, and ^∆∆∆∆^p < 0.0001 versus the 12 h group

We found that the apoptotic rate of L02 cells was markedly increased after prolonged culture in serum deprivation and hypoxia, as demonstrated by Annexin V and PI double staining (Fig. 3a, b). Cell viability was inversely correlated to the time of culture and reached statistical significance at 18 h (Fig. 3b). Moreover, the lactate dehydrogenase (LDH) levels of L02 cells increased during the induction of apoptosis (Fig. 3b). These observations confirmed that this cellular model mimics the hepatocellular impairment after brain death, with an important features of elevated apoptosis.

We also found that the activity of PP2A in L02 cells was remarkably increased over time under serum deprivation and hypoxia, while the opposite was observed for the activity of Akt (Fig. 3c). Although western blotting failed to show change in total Akt and PP2A protein level, Akt phosphorylation at Ser473 decreased during the time of hypoxia exposure, consistent with the diminishing Akt activity (Fig. 3d). For other known targets of PP2A, such as p38, JNK and ERK, the content of total protein as well as phosphorylated forms seemed not to be affected (Fig. 8). These results implied that PP2A could be involved in the regulation of hepatocytes apoptosis after brain death, possibly through dephosphorylation and inhibition of Akt activity.

### Hypoxia and ischemia (mimic BD) induced Akt inactivation is directly regulated by PP2A activation in vitro

**Fig. 4:**
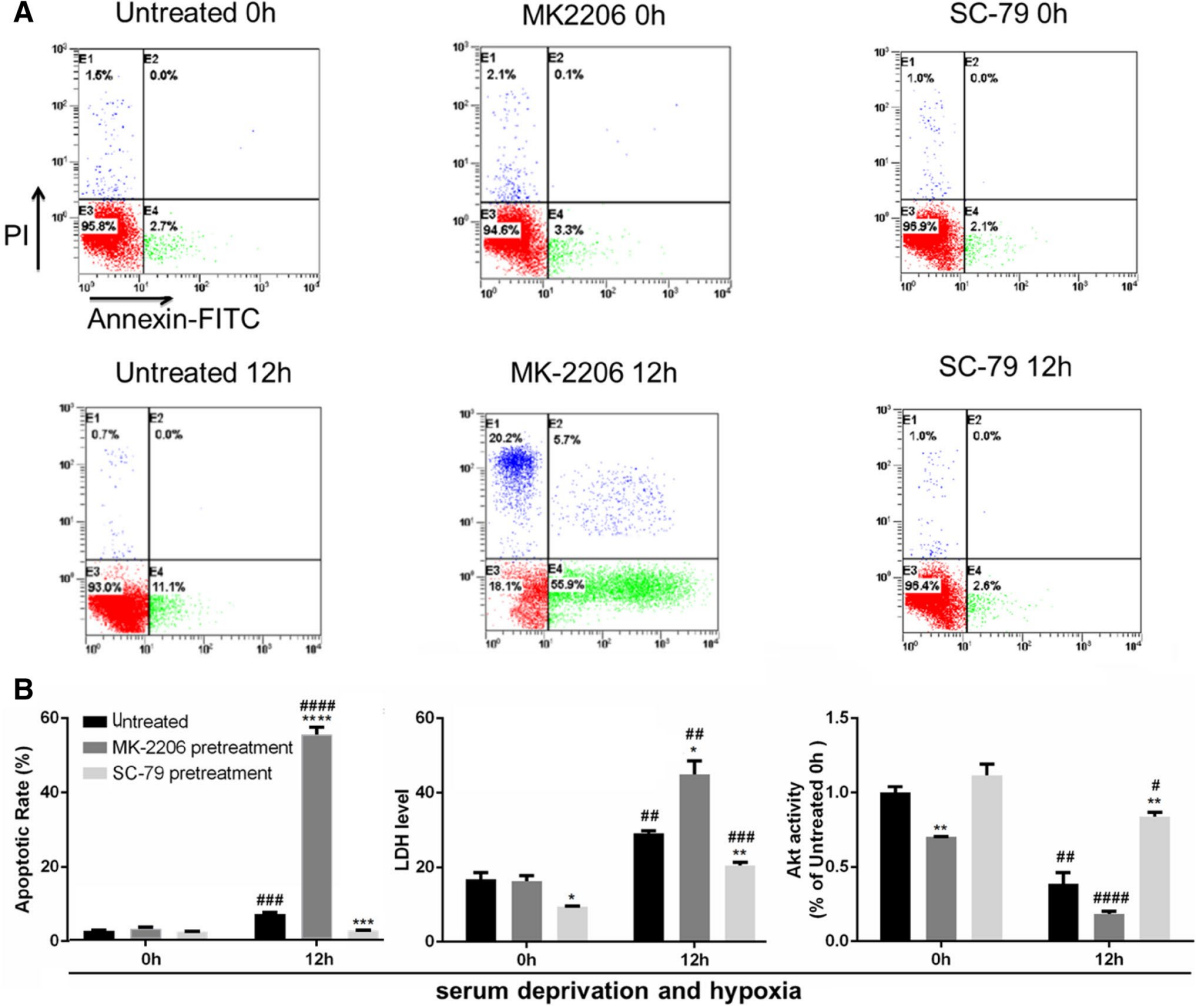
Upon MK-2206 (Akt inhibitor) pretreatment, apoptosis and cell damage were aggravated in L02 cells exposed to serum deprivation and hypoxia; while all the phenotypes were rescued to an extent upon SC-79 (Akt activator) pretreatment. MK-2206 was used at a concentration of 10 nM and SC-79 at a concentration of 5 μM. The cells were pretreated with the compounds for 60 min before exposure to serum deprivation and hypoxia. **a** Representative FACS plots of apoptosis detection were shown. **b** Left: quantitative analysis of the apoptotic rate. Middle: LDH measurement; the unit: U/L. Right: the Akt activity measurement normalized to the untreated 0 h group. All data were expressed as the mean ± SEM. n = 3 for each group in each measurement. *p < 0.05, **p < 0.01, ***p < 0.001 and ****p < 0.0001 versus untreated cells at the same time; ^#^*p* < 0.05, ^##^*p* < 0.01, ^###^*p* < 0.001, and ^####^*p* < 0.0001, 12 h versus 0 h, with the same treatment

As hepatocytes underwent massive necrosis after 18 h of serum deprivation and hypoxia, we looked at the apoptotic rate of these cells after 12 h. The treatment with AKT inhibitor (MK-2206) or activator (SC-79) [23, 31] correspondingly changed Akt activity (Fig. 4b), and respectively deteriorated or ameliorated the impairment of L02 cells under serum deprivation and hypoxia, as indicated by apoptotic rate and LDH level (Fig. 4a, b). It proved that Akt could inhibit hepatocyte apoptosis and attenuate liver cell damage upon the deficiency of nutrient and oxygen.

**Fig. 5:**
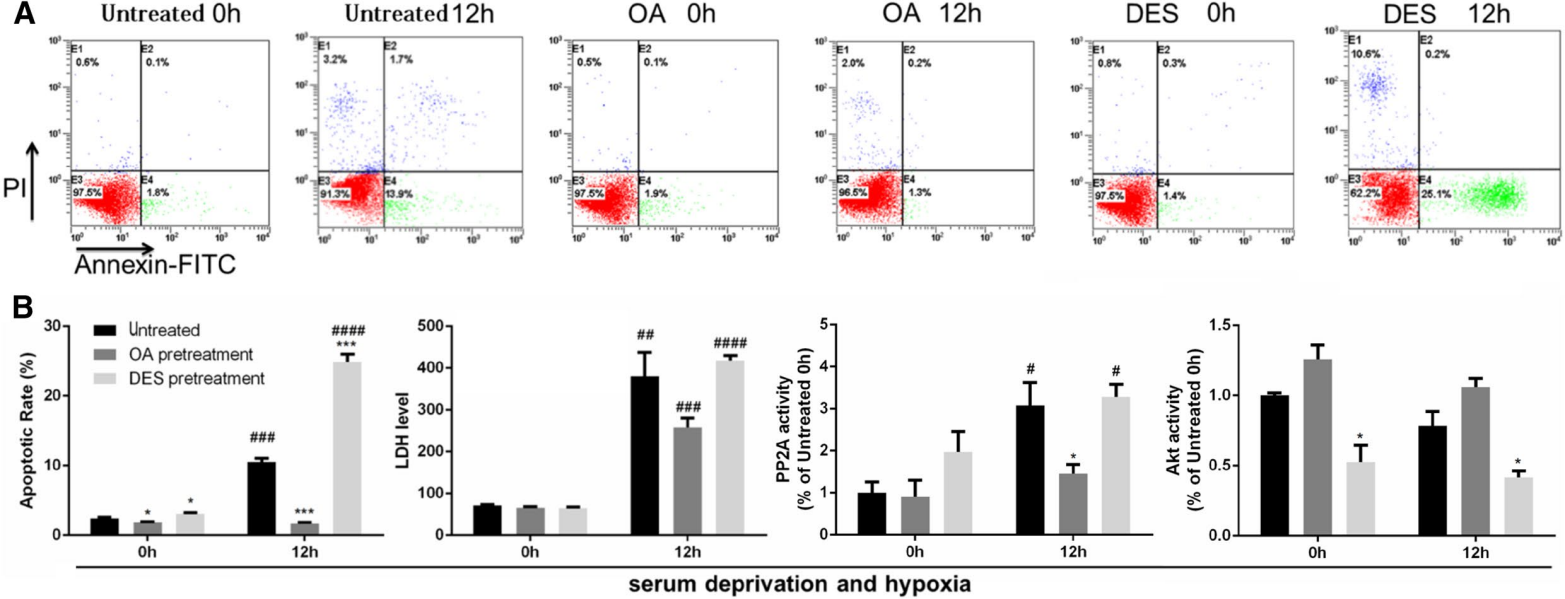
Upon okadaic acid (OA) (PP2A inhibitor) pretreatment, apoptosis and cell damage were reversed to an extent in L02 cells exposed to serum deprivation and hypoxia, so was Akt inactivation; while all the phenotypes became more severe upon D-erythro-S (DES) (PP2A activator) pretreatment. OA was used at a concentration of 10 nM and DES at a concentration of 5 μM. The cells were pretreated with the compounds for 60 min before exposure to serum deprivation and hypoxia. **a** Representative FACS plots of apoptosis detection were shown. **b** From left to right: Quantitative analysis of the apoptotic rate; LDH measurement, the unit: U/L; The PP2A activity and Akt activity measurement normalized to the untreated 0 h group. All data were expressed as the mean ± SEM. n = 3 for each group in each measurement. *p < 0.05, and ***p < 0.001 versus untreated cells at the same time; ^#^*p* < 0.05, ^##^*p* < 0.01, ^###^*p* < 0.001, and ^####^*p* < 0.0001, 12 h versus 0 h, with the same treatment

Furthermore, experiments with PP2A inhibitor okadaic acid (OA) [32] or its activator D-erythro-S (DES) [33] showed that altering PP2A activity would inversely affect Akt activity (Fig. 5b), demonstrating the role played by PP2A in repressing Akt signalling. On these grounds PP2A activation would induce hepatocyte apoptosis and damage (Fig. 5a, b) under poor nutrition and hypoxic condition. We considered that this mechanism is responsible for in vivo hepatocellular impairment in DBD.

**Fig. 6:**
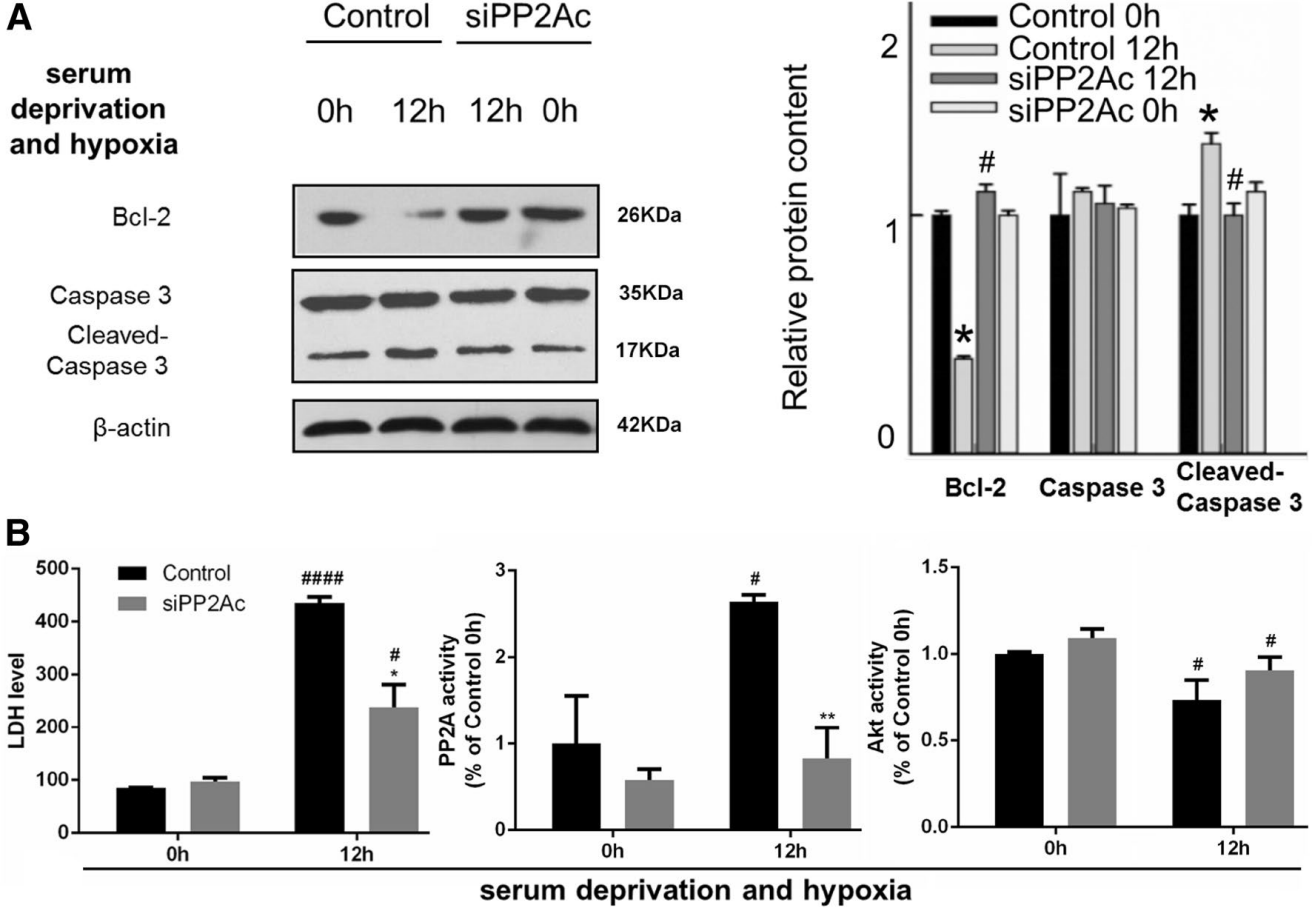
After PP2Ac knockdown, apoptosis and cell damage were reversed to an extent in L02 cells exposed to serum deprivation and hypoxia, so was Akt inactivation. L02 cells were transfected with GFP-siPP2Ac plasmid (the siPP2Ac group) or GFP-vector (the control group) for 48 h. Then cells were serum deprived and cultured under hypoxic conditions. **a** Left: representative images of western blots for apoptotic markers Bcl-2, caspase-3 and cleaved caspase 3. Right: semi-quantitative analysis with β-actin as an internal control. The results were normalized to the control 0 h group. **b** Left: LDH measurement; the unit: U/L. Middle and right: the PP2A or Akt activity measurement normalized to the control 0 h group. All data were expressed as the mean ± SEM. n = 3 for each group in each measurement. *p < 0.05 and **p < 0.01 versus control cells at the same time; ^#^*p* < 0.05 and ^####^*p* < 0.0001, 12 h versus 0 h, with the same transfection

**Fig. 7:**
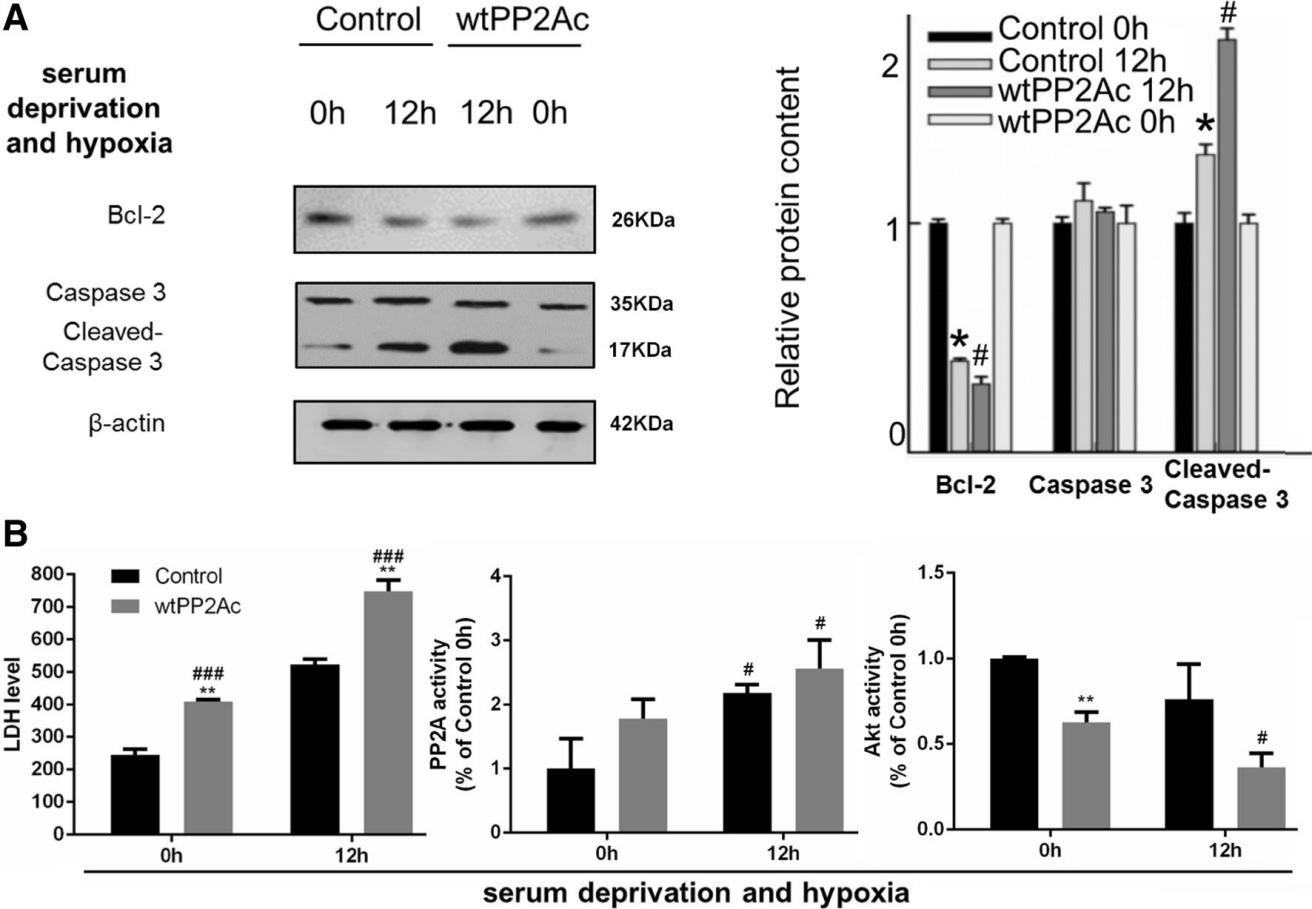
After PP2Ac overexpression, apoptosis and cell damage became more severe in L02 cells exposed to serum deprivation and hypoxia, so did Akt inactivation. L02 cells were transfected with RFP-wtPP2A plasmid (the wtPP2A group) or RFP-vector (the control group) for 48 h. Then cells were serum deprived and cultured under hypoxic conditions. **a** Left: representative images of western blots for apoptotic markers Bcl-2, caspase-3 and cleaved caspase 3. Right: semi-quantitative analysis with β-actin as an internal control. The results were normalized to the control 0 h group. **b** Left: LDH measurement; the unit: U/L. Middle and right: the PP2A or Akt activity measurement normalized to the control 0 h group. All data were expressed as the mean ± SEM. n = 3 for each group in each measurement. *p < 0.05 and **p < 0.01 versus control cells at the same time; ^#^*p* < 0.05 and ^###^*p* < 0.001, 12 h versus 0 h, with the same transfection

**Fig. 8:**
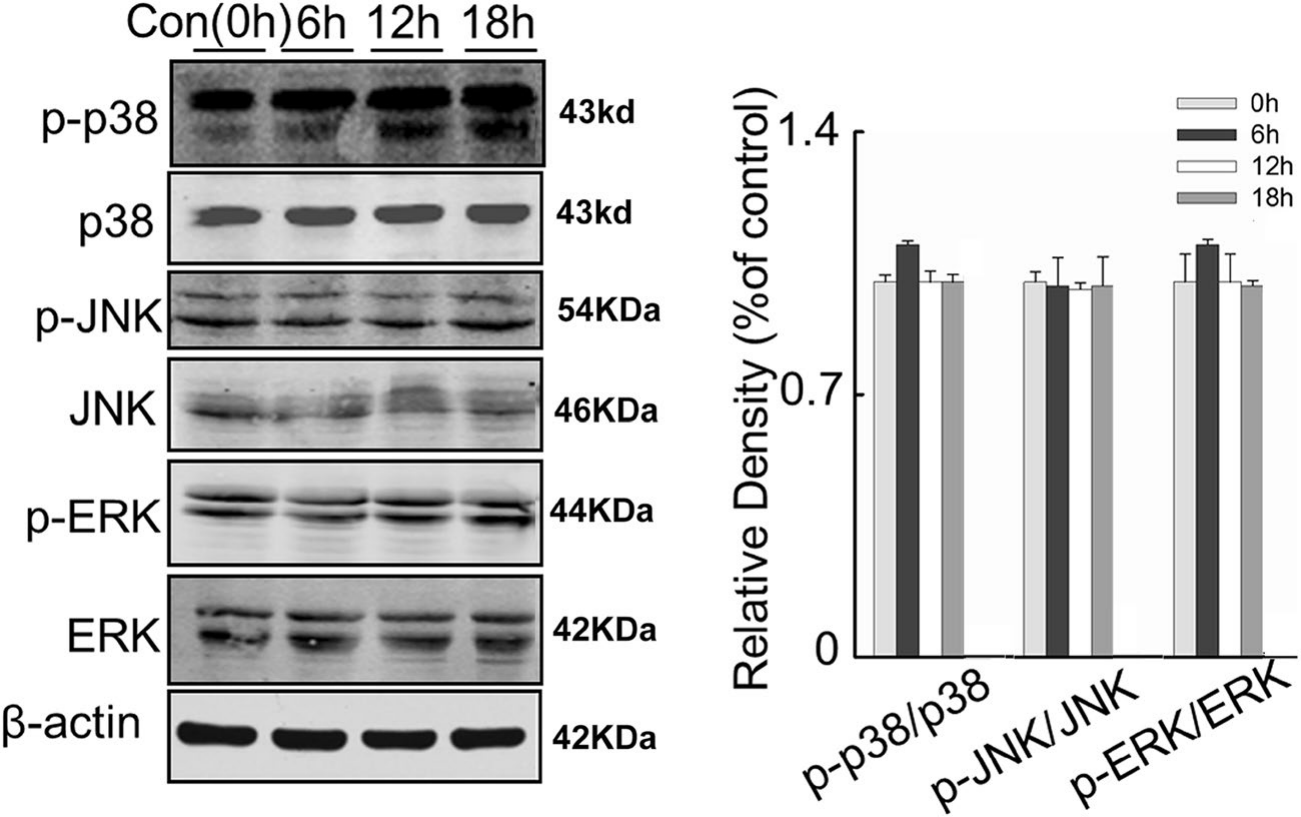
L02 cells were serum deprived and cultured under hypoxic conditions and the cell signaling markers were measured at 0, 6, 12 and 18 h of that exposure. Left: representative western blots of the expression of the proteins p38, JNK, ERK and their phosphorylated counterparts. Right: semi-quantitative analysis of the expression of the proteins using β-actin as an internal control. All data were expressed as the mean ± SEM. n = 3 for each group in each measurement

**Fig. 9:**
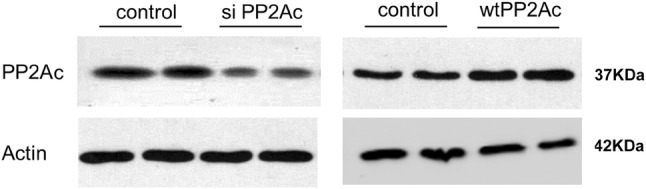
The validation of the transfection efficiency of the wtPP2Ac plasmid and the siPP2Ac sequence in L02 cells using western blotting

To further verified the regulatory role of PP2A in hepatocyte apoptosis post brain death, we knockdown PP2A by using a GFP-tagged *pp2ac* siRNA (siPP2Ac) or we over-expressed PP2A by transfecting aRFP-tagged wildtype form of PP2Ac (wtPP2Ac) L02 cells. The transfection efficiencies of siPP2Ac or wtPP2A were confirmed by western blot analysis of PP2Ac (Fig. 9). PP2Ac knockdown significantly decreased cleaved Caspase-3 and increased Bcl-2 levels and increased the activity of Akt (Fig. 6). Overexpression of PP2Ac showed the opposite effect on cleaved Caspase-3, Bcl-2 and the activity of Akt (Fig. 7). These results further confirmed the involvement of PP2Ac in the regulation of apoptosis in liver cells under hypoxic condition through inhibiting Akt (Figs. 8, 9).

## Discussion

In the present study, we revealed the involvement of PP2A in the induction of apoptosis in liver cells after brain death, by negatively regulating Akt activity. Suffering from undernourished and hypoxic conditions, the hepatocytes showed an increase in PP2A activity, which afterwards suppressed Akt activity but had little effect on the p38, JNK or ERK signaling. The inhibition of Akt activity may be the result of decreased phosphorylation at Ser473. Apoptosis level in the damaged hepatocytes were promoted as a consequence of such “PP2A activation-Akt inhibition” axis, and pharmaceutical targeting of PP2A or Akt could correspondingly worsen or attenuate the hepatocellular damage.

Brain death (BD) is defined as the irreversible cessation of all brain activities, including that in the brain stem. Clinical and experimental studies have shown that BD is a complex pathological process that damage organ morphology and function [34–36]. The physiological changes that occur after BD include hemodynamic changes, blood coagulation, endocrine and/or electrolyte disorders. Several studies have shown that grafts from BD donors have a lower short- and long-term posttranslational effectiveness and higher incidences of primary graft non-function [37] and higher incident of acute rejection [38], in comparison with that from living donors. Despite these crucial observations, the exact etiology of the poor quality of donor-liver function has not been fully examined.

Novitzky et al. reported that BD leads to hemodynamic changes, characterized by a dramatic increase in both systolic and diastolic blood pressure [39]. These effects lead to tachycardia and the cardiac output is increased by 30%, whereas a 50% increase is noted in the peripheral vascular resistance [39]. The blood flow in the portal vein and the hepatic tissue has been reported to be approximately 40% of that corresponding to the hypotensive state in the absence of brain death [8] leading to hepatic hypoxia. In the present study, hypoxia and ischemia were induced at the cellular level for the time periods of 0 h, 6 h, 12 h and 18 h, in order to mimic hepatic ischemia induced by brain death.

Several reports showed that the increased rate of apoptosis is one of the main factors that lead to poor liver graft quality [10, 12, 40, 41]. In our previous study, we confirmed that the increased apoptosis is the most important factor contributing to decreasing liver functions under the brain death conditions [28]. Intervention at the level of the apoptotic pathway is likely to be a promising target for drug development, in terms of improving the quality of liver graft to decrease the incident of primary graft non function and acute rejection.

Previous studies have indicated that brain death induces liver donor cell apoptosis by various signaling pathways. Brain death can change the inflammation response, which releases inflammatory mediators and promotes apoptosis [5]. In addition, it can contribute directly to the activation of caspase 3 leading to apoptosis [38]. HO-1, which has strong anti-oxidant activity, can improve long-term brain dead graft survival [40]. Thus, all evidences have pointed to brain dead induced cell apoptosis as the cause of poor graft quality.

In the current study, with the use of multiple biochemical and pharmacological approaches, we found that brain death activated PP2A and inactivated Akt in the liver. This finding was further verified with brain-death-mimic cell culture environment, in which hepatocyte apoptosis was induced. In this case PP2A activity increased and subsequently dephosphorylates Akt on ser473. PP2A is considered as a tumor suppressor enzyme that affects multiple signaling cascades such as the Ras, PI3 K, Akt and PKC pathways that have been implicated in various cancers [42]. The activation of PP2A is a common event that is noted in several cancers that occurs as a result of the perturbed regulation of the cell growth and survival [43]. Many reports showed that PP2A is activated with cAMP/PKA [44, 45]; hypoxia can induce cAMP/PKA activation through HIF transcription factor [46]. Therefore, we speculated that enhanced PP2A activity in our brain death in vivo and in vitro model was due to the induction of hypoxia after brain death. With regard to Akt, PP2A has been shown to directly interact with this protein via the subunit B55 alpha [47]. PP2A inactivates Akt by dephosphorylation and in hepatoma cancers the reduced expression of PP2A B55 alpha subunit results in the increased activation of Akt and consequently increased growth and proliferation [48]. Previous studies demonstrated that the overexpression of eIF3I interacted with and activated the oncogenic Akt1 by preventing the PP2A-mediated dephosphorylation of Akt-1 in human hepatocellular carcinoma [24]. REDD1 enhances protein phosphatase 2A-mediated dephosphorylation of Akt, in order to repress mTORC1 signaling in 293T cells [25]. For the first time in the current study we demonstrated that the down regulation of Akt activity by PP2A results in apoptosis in liver cells in the brain-dead donors or in a similar in vivo brain death model.

In conclusion, the findings presented here disclose one of the underlying mechanisms for hepatocellular impairment after brain death, and imply potential targets for reducing hepatocyte apoptosis and improving hepatic graft quality, through PP2A inhibition or Akt activation. Studies in this field will help to prolong organ graft survival and lower the incidence of primary graft non-function or acute rejection.

## Materials and methods

### Clinical DBD samples

The study was approved by the ethics committee of the Zhongnan Hospital of Wuhan University. All patients or their legal representatives provided a written informed consent.

From February 2013 to March 2016, a total of 20 samples of DBD liver tissues were respectively collected from 20 donors at 2 h, 6 h or 12 h post brain death (Table S1) in the Zhongnan Hospital of Wuhan University, Wuhan, China. The DBD liver tissues were extracted without perfusion. The tissues were suitable for transplantation, i.e., without any lesions, cirrhosis, fatty liver disease, etc. The preservation solution was UW solution.

The liver tissues were homogenized in 10 volumes (mL/g wet tissue) homogenate buffer containing 50 mM Tris–HCl, pH 7.0, 0.5 mM Phenylmethylsulfonyl fluoride (PMSF), 2.5 mM ethylenediaminetetraacetic acid (EDTA), 2.5 mM ethylene glycol tetra acetic acid (EGTA), and 1:1000 protease inhibitor cocktail (Sigma-Aldrich Co., LLC., St. Louis, MO, USA). The tissue lysates were sonicated, and centrifuged at 16,000×*g* for 10 min. The supernatants were used for the evaluation of the PP2A and Akt activity assays.

### Protein extraction and iTRAQ mass spectrometry analysis

Liver samples after brain death for 2 h and 12 h were collected. Each sample (100 mg of protein) was digested with SDT solution and labeled with iTRAQ reagents (Applied Biosystems) according to the manufacturer’s protocol. Subsequently, the labeled peptides were mixed equally and separated by 1260 Infinity HPLC (Agilent Technologies), followed by nano liquid chromatography tandem mass spectrometry using the Hybrid Quadrupole-Orbitrap mass spectrometer (Q-Exactive; Thermo Fisher Scientific) equipped with a nano-UPLC RSLC Ultimate 3000 (Dionex). Both peptide identification and quantitation were performed in an overall workflow in Proteome Discoverer software (version 1.4; Thermo Fisher Scientific) and searched against the UniProt human canonical sequence protein database (October 7, 2011; 56,869 entries) using Mascot search engine (version 2.4). For protein identification, 95% confidence was used. For quantitation and further validation experiments, all reported data were based on 95% confidence for protein identification as determined by Proteome Discoverer (Unique peptide > 1)

### Animals

The study was carried out in strict accordance with the recommendations of the Guide for the Care and Use of Laboratory Animals of the National Institutes of Health. The protocol was approved by the Committee of the Ethics of Animal Experiments of Wuhan University 2014. All surgery was performed under sodium pentobarbital anesthesia, and all efforts were made to minimize suffering.

A total of 40 male New Zealand rabbits (weight 2800–3000 g, 3 months old) were obtained from the Center for Animal Experiments and ABSL-3 Laboratory of the Wuhan University (Wuhan, China). All animals were kept under standard laboratory conditions: 12 h of light and 12 h of darkness; lights were turned on at 6:00 a.m.; the temperature was kept at 22 ± 2.0 °C; and water and food were ad libitum.

The DBD model was established in 30 rabbits as described previously [28, 29]. The rabbits were deeply anesthetized with pentobarbital sodium at a dose of 40 mg/kg and then placed on the operating table. The femoral artery and vein cannulation, xiphoid separation and tracheal intubation, burr hole and catheter placement were carried out. The vital signs of the rabbits including electrocardiogram, blood pressure, respiratory functions, and electroencephalogram were monitored using a biological functional system, a rodent ventilator, and an intelligent temperature control instrument (Thai Union Technology, Co., Ltd., Chengdu, China). The intracranial pressure was increased as required until the occurrence of brain death. A total of ten rabbits were sacrificed respectively at 2 h, 4 h, and 8 h, following brain death.

The remaining ten rabbits received deep anesthesia without an increase in the intracranial pressure, and they were sacrificed 2 h following sham operation.

The liver tissues obtained from the animals were then processed as methods described for clinical samples, and used for the PP2A/Akt activity assays. Besides, after sonication, a part of tissue lysates was added with equal volume of phosphate inhibitor mixture (2.0 mM Na_3_VO_4_ and 100 mM NaF, pH 7.0) and stored at − 80 °C for Western blotting.

### Cell culture

Human liver cell line L02 was obtained from Kunming Institute of Zoology (Chinese Academy of Sciences, Kunming, China). The cells were grown to 70–80% confluence in six-well culture plates in Dulbecco’s Modified Eagle’s medium (Gibco (Life Technologies), CA, USA) supplemented with 10% fetal bovine serum (FBS), 100 units/mL penicillin, and 0.1 mg/mL streptomycin.

The L02 cells were exposed to small molecule treatment or transfected with specific plasmids (see detailed description below). Then the cells were cultured in serum-free DMEM/low glucose medium (HyClone, USA) and placed in a tri-gas incubator (thermo fisher 3131, Waltham, MA USA) which can control the oxygen concentration at 1%, to make ischemic and hypoxic conditions mimicking adverse environment of liver in BD donors [30]. The specific processing times were indicated in the corresponding figures.

After different treatments, some of cells were used for flow cytometry analysis, cell viability analysis and lactate dehydrogenase (LDH) measurement. The remaining cells were harvested and suspended in buffer containing 2.0 mM EGTA, 0.5 mM PMSF, 5 mM EDTA, 150 mM NaCl, 50 mM Tris–HCl (pH 7.4), 1.0% Triton X-100, and a protease inhibitor cocktail (1:200). The cell lysates were sonicated and divided into three parts. The first part was mixed with equal volume of phosphate inhibitor mixture (2.0 mM Na_3_VO_4_ and 100 mM NaF, pH 7.0) and stored at − 80°C for Western blotting. The rests were centrifuged at 16,000×*g* for 10 min, and the supernatants were used for PP2A and Akt activity assay.

### Small molecule pretreatment

L02 cells were pretreated with specific concentrations of small molecules at 60 min time periods. The Akt inhibitor MK-2206 (Selleckchem, USA) and the PP2A inhibitor OA (Santa cruz, USA) were incubated with L02 cells at a concentration of 10 nM. The Akt activator SC79 (TOCRIS bioscience, Missouri, USA) and the PP2A activator DES (San Diego, CA, USA) were incubated with L02 cells at concentrations of 4 μg/mL and 5 μM, respectively.

### Cell transfection

The plasmids carrying the EGFP-siPP2Ac (a small interfering RNA construct designed specifically for *pp2ac*) and the DsRed-wild-type (wt) PP2Ac were kindly provided by Professor Jianzhi Wang (Tongji Medical College, HuaZhong University of Science and Technology, Wuhan, China). L02 cells were transfected with EGFP-siPP2A or DsRed-wtPP2Ac plasmids using Lipofectamine 2000 according to the manufacturer’s instruction (Invitrogen, San Diego, CA, USA). The plasmids that contained solely fluorescent proteins were used as the controls. Following 48 h of transfection, the cells were used for further analyses.

### Flow cytometry analysis

The cellular apoptosis and necrosis were assessed using the Annexin V-FITC apoptosis kit (Franklin Lakes, NJ, USA) according to the manufacturer’s instructions. The cells were digested with trypsin, centrifuged at 1000×*g* for 5 min, and incubated with 5 μL Annexin V and 5 μL PI for 10 min at room temperature in the dark. The cells from each sample were then identified using a FacsCalibur flow cytometer (BD Biosciences). The data were analyzed using CELLQuest software (BD). The experiments were repeated at least three times.

### Cell viability analysis

The cell viability was assessed with the Cell Counting Kit-8 detection kit (CCK8) (Dojindo Laboratories, Kumamoto, Japan) according to the manufacture’s protocol. A total of 1 × 10^5^ cells were cultured in 96-well plates. The cells were performed with different treatments, and then 100 μL 10% CCK8 solution was added in each well. The samples were incubated at 37 °C for 1 h. The absorbance was measured at 450 nm using a microplate reader (VersaMax, Molecular Devices, USA). The experiments were repeated at least three times.

### Lactate dehydrogenase (LDH) measurement

The cell medium was collected for the LDH measurement using the AU5400 Clinical Chemistry System (beckman coulter, USA), and the results were expressed in the unit of U/L. The experiments were repeated at least three times.

### Western blotting

The liver tissue lysates (from clinical samples or modeling rabbits) or the L02 cell lysates were electrophoresized in 10% SDS-polyacrylamide gel and the proteins were transferred to nitrocellulose membranes. The membranes were incubated with specific antibodies. Primary antibodies against: pSer473 Akt, Bcl-2, Caspase 3, Cleaved-Caspase 3, p38, p-p38, JNK, p-JNK, ERK, p-ERK were from cell signaling (Danvers, MA, USA); total Akt was from Proteintech (Chicago, UK); total PP2Ac was from Millipore (Billerica, MA, USA); β-actin was from Abcam (Cambridge, UK). Secondary antibodies conjugated to I IRDye™ 800CW were from Licor Biosciences (Lincoln, NE, USA). The results were visualized using the Odyssey Infrared Imaging System (LI-COR Biosciences, Lincoln, NE, USA).

### PP2A/Akt activity assay

The supernatants of the liver tissue lysates (from clinical samples or modeling rabbits) or L02 cell lysates were prepared as described above. The activity of PP2A in the supernatants was detected using the phosphatase kit V2460 (Promega, Madison, WI, USA) according to the manufacturer’s protocol, with the results expressed as nmol phosphorus/mg/min. The activity of Akt was detected using the colorimetric method quantity detecting kit (Genmed Scientifics Inc., Arlington, MA, USA) according to the manufacturer’s protocol, with the results expressed as μmol NADH/mg/min.

### Statistical analysis

The data are expressed as the mean ± standard error of measurement (SEM) and analyzed using SPSS 16.0 (SPSS Inc., Chicago, IL, USA). The one-way analysis of variance (ANOVA) procedure followed by the Fisher’s Least Significant Difference post hoc test (LSD) and Student’s *t* test were used to determine the differences among the groups. The statistical differences were considered significant if the *p* value was < 0.05.

## Electronic supplementary material

Below is the link to the electronic supplementary material.

Supplementary material 1 (DOCX 16 kb)
