# Supplementary material for: PP2Ac upregulates PI3K-Akt signaling and induces hepatocyte apoptosis in liver donor after brain death
Source: Apoptosis. 2019 Oct 11;24(11):921–33. doi: 10.1007/s10495-019-01570-8 (PMC6823653; doi:10.1007/s10495-019-01570-8)
Supplement: Supplementary file 1 — Supplementary material 1 (DOCX 16 kb) [file 10495_2019_1570_MOESM1_ESM.docx]

**Supporting information**

**Table 1** Characteristics of the brain dead donors

| NO. | Age | Sex | Blood type | reasons for brain death | Time of  brain death |
| --- | --- | --- | --- | --- | --- |
| 1 | 22 | male | O | subarachniod hemorrhage | 2h |
| 2 | 32 | male | A | glioma | 2h |
| 3 | 21 | female | A | glioma | 2h |
| 4 | 26 | male | B | brain trauma (traffic accident) | 2h |
| 5 | 45 | male | O | subarachniod hemorrhage | 2h |
| 6 | 21 | female | O | brain trauma(traffic accident) | 2h |
| 7 | 48 | male | A | cerebral infarction | 2h |
| 8 | 56 | male | A | subarachniod hemorrhage | 2h |
| 9 | 42 | male | O | brain trauma(traffic accident) | 2h |
| 10 | 65 | male | O | cerebral infarction | 6h |
| 11 | 45 | male | O | brain trauma(traffic accident) | 6h |
| 12 | 42 | male | O | brain trauma(traffic accident) | 6h |
| 13 | 48 | male | AB | subarachniod hemorrhage | 6h |
| 14 | 37 | female | AB | brain trauma(traffic accident) | 6h |
| 15 | 45 | male | A | subarachniod hemorrhage | 12h |
| 16 | 41 | male | A | cerebral infarction | 12h |
| 17 | 17 | male | AB | brain trauma(traffic accident) | 12h |
| 18 | 39 | male | B | brain trauma(traffic accident) | 12h |
| 19 | 41 | male | O | glioma | 12h |
| 20 | 54 | female | O | brain trauma(traffic accident) | 12h |
